# Exploring Hirsutism: Epidemiology, Associated Endocrinal Abnormalities, and Societal Challenges in GCC—A Narrative Review

**DOI:** 10.3390/ijms26125575

**Published:** 2025-06-11

**Authors:** Mohamed Anas Patni, Rajani Dube, Subhranshu Sekhar Kar, Biji Thomas George, Manjunatha Goud Bellary Kuruba, Suresh Kumar Srinivasamurthy, Abdalla Ahmed Eldaw Elamin

**Affiliations:** 1 Department of Community Medicine, RAK College of Medical Sciences, RAK Medical and Health Sciences University, Ras Al Khaimah P.O. Box 11172, United Arab Emirates; mohamedanas@rakmhsu.ac.ae; 2 Department of Obstetrics and Gynaecology, RAK College of Medical Sciences, RAK Medical and Health Sciences University, Ras Al Khaimah P.O. Box 11172, United Arab Emirates; 3 Department of Paediatrics and Neonatology, RAK College of Medical Sciences, RAK Medical and Health Sciences University, Ras Al Khaimah P.O. Box 11172, United Arab Emirates; subhranshu.kar@rakmhsu.ac.ae; 4 Department of General Surgery, RAK College of Medical Sciences, RAK Medical and Health Sciences University, Ras Al Khaimah P.O. Box 11172, United Arab Emirates; biji@rakmhsu.ac.ae; 5 Department of Biochemistry, RAK College of Medical Sciences, RAK Medical and Health Sciences University, Ras Al Khaimah P.O. Box 11172, United Arab Emirates; manjunatha@rakmhsu.ac.ae; 6 Department of Pharmacology, RAK College of Medical Sciences, RAK Medical and Health Sciences University, Ras Al Khaimah P.O. Box 11172, United Arab Emirates; suresh@rakmhsu.ac.ae; 7 Department of Anatomy, RAK College of Medical Sciences, RAK Medical and Health Sciences University, Ras Al Khaimah P.O. Box 11172, United Arab Emirates; abdalla@rakmhsu.ac.ae

**Keywords:** hirsutism, polycystic ovary disease, Cushing disease, congenital adrenal hyperplasia, idiopathic hirsutism, molecular, gene, pathophysiology, social stigma, Gulf Cooperation Council

## Abstract

Hirsutism, characterized by excessive terminal hair growth in androgen-sensitive areas, presents significant medical and psychosocial challenges in Gulf Cooperation Council (GCC) countries. This narrative review explores the epidemiology, endocrine factors, molecular basis of pathophysiology, cultural influences, and management approaches to hirsutism within the GCC. Regional factors such as consanguinity, rising obesity rates, and lifestyle habits contribute to a higher prevalence of hirsutism and related endocrine disorders, particularly polycystic ovary syndrome (PCOS). Cultural stigmas surrounding body hair further delay diagnosis and treatment, compounding psychological distress. The review examines the role of androgen excess, genetic susceptibility, and emerging molecular insights, including epigenetic dysregulations. Diagnostic limitations and the need for region-specific screening tools are discussed, alongside the current reliance on pharmacological, cosmetic, and traditional therapies. Public health initiatives targeting stigma reduction and early detection are emphasized. Future recommendations include culturally tailored research, enhanced public awareness, and the adoption of advanced diagnostic strategies to improve patient outcomes. This review aims to guide healthcare practices and inform policy development for the better management of hirsutism in the GCC context.

## 1. Introduction

Hirsutism is a disorder with significant medical, psychological, and cultural ramifications that is typified by excessive terminal hair growth in androgen-dependent areas [1]. It is most frequently linked to hyperandrogenism brought on by endocrine conditions such as Cushing’s syndrome, congenital adrenal hyperplasia, and polycystic ovary syndrome (PCOS) [2,3]. About 5–10% of women worldwide suffer from hirsutism, with regional variations in frequency caused by lifestyle, environmental, and genetic variables [4]. Despite new data indicating a larger frequency of the ailment, hirsutism is still a relatively understudied problem, with few comprehensive studies conducted in Gulf Cooperation Council (GCC) member nations.

Higher rates of Hirsutism in GCC countries can be attributed to unique regional factors such as the practice of consanguinity, increasing rates of obesity, and specific dietary habits that characterize the lifestyles in these areas [5]. Cultural standards, societal expectations, and personal identity are all impacted by the disease, which goes beyond a simple medical diagnosis. Women with hirsutism endure severe stigma and psychological hardship in addition to health issues in the GCC, where hairlessness is highly valued according to beauty standards [6,7,8]. GCC underreporting and delayed medical care are made worse by cultural taboos around talking about reproductive health [9,10]. These difficulties emphasize the necessity of a thorough comprehension of the illness in light of the GCC’s distinct sociocultural and medical setting [11]. Addressing these problems calls for focused public health campaigns that dispel the stigma attached to reproductive health issues while also educating communities about hirsutism and its effects.

The purpose of this review is to examine the various facets of hirsutism in GCC nations, with an emphasis on its prevalence, cultural attitudes, endocrine foundations, diagnostic methods, and management techniques. This study aims to enhance patient outcomes, influence public health policy, and direct future research in the area by finding gaps in the literature and synthesizing them.

## 2. Methods

### 2.1. Search Strategy

For this review, we conducted a narrative literature search to explore the prevalence, endocrinal factors, cultural influences, and management of hirsutism in GCC countries. To ensure a comprehensive yet focused literature search, we used three major electronic databases—PubMed, Scopus, and Google Scholar—as they offer extensive coverage of medical and clinical research. A structured search strategy was applied, using Medical Subject Heading (MeSH) terms and Boolean operators to identify relevant articles. The search targeted three primary themes: hirsutism and hyperandrogenism; endocrinal, genetic, and metabolic factors, and epidemiology and cultural perspectives in the GCC. Keywords such as “hirsutism,” “hyperandrogenism,” “polycystic ovary syndrome (PCOS),” “insulin resistance,” “GCC,” and specific country names (e.g., Saudi Arabia, UAE, Oman) were combined strategically to refine the results. The keywords were combined using Boolean operators to construct the final search string:

(“Hirsutism” OR “Hyperandrogenism” OR “PCOS” OR “Metabolic syndrome” OR “Insulin resistance”) AND (“GCC” OR “Saudi Arabia” OR “UAE” OR “Oman” OR “Kuwait” OR “Qatar” OR “Bahrain”)

To ensure quality and relevance, we included peer-reviewed studies published in English within the last 10 years (2014–2024), prioritizing original research, systematic reviews, and meta-analyses focusing on hirsutism and related endocrinal disorders in the GCC or similar populations. Studies that did not directly address hirsutism, were unrelated to the GCC region, or were not available in full-text English versions were excluded. After an initial search, we screened titles and abstracts to eliminate duplicates and non-relevant studies, and the full texts of selected articles were reviewed to assess their methodological rigor and contribution to the topic.

To manage references and ensure proper organization, we used Zotero 6, a citation management tool, which allowed for the efficient sorting and retrieval of selected studies. The findings were then synthesized to present a coherent overview of hirsutism’s underlying endocrinal mechanisms, its prevalence in the GCC, cultural attitudes, and available treatment approaches. Given the limited data specifically from GCC countries, we also incorporated findings from neighboring regions with similar genetic, dietary, and environmental influences, such as the Middle East and South Asia.

**Figure 1:**
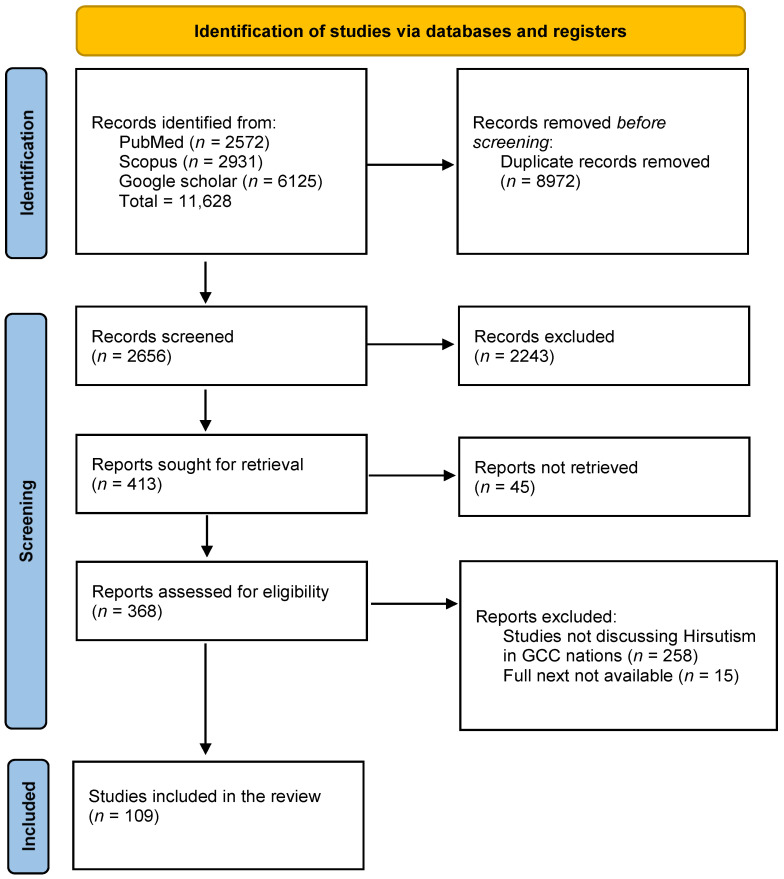
PRISMA flow diagram for selection of studies.

This study made it possible to analyze 109 scientific articles published—preferably in last 15 years—related to Hirsutism in GCC nations. The PRISMA flow diagram of each stage of the article selection process is described in the flowchart shown in Figure 1.

### 2.2. Data Collection

Four authors (R.D., S.S.K., M.A.P., B.T.G.) reviewed all titles independently. The potential relevance of the studies to be included for review was agreed upon by discussion on a regular basis. Selected titles and abstracts were further screened between studies to reject the overlap of cases. Full-text copies of the selected papers were obtained, and the relevant data were extracted. The decision on exclusion or inclusion was made by discussion. The risk of bias was not assessed due to the nature of the studies.

## 3. Results and Discussion

### 3.1. Prevalence and Epidemiology

About 4–11% of women worldwide suffer from hirsutism, a disorder characterized by excessive terminal hair growth in androgen-sensitive regions [12]. The exact prevalence is largely unknown because of the paucity of research conducted specifically in the GCC. However, current research suggests that genetic, environmental, and lifestyle variables may contribute to the region’s higher prevalence of hirsutism and its most common underlying cause, PCOS [13,14,15]. The incidence of PCOS, a major cause of hirsutism, has been somewhat clarified by studies conducted in Saudi Arabia, the United Arab Emirates, and Oman. For instance, a cross-sectional study in Riyadh, Saudi Arabia, revealed a prevalence of PCOS of 28%, which is much higher than the 6–20% global average [16,17]. Similarly, studies conducted in the United Arab Emirates show a high prevalence of metabolic syndromes, which frequently coexist with PCOS and lead to hirsutism and hyperandrogenism [18,19]. An important contributing element to androgen excess, insulin resistance, is made worse by the growing obesity rates in GCC nations [20,21].

Findings from the GCC are consistent with comparative data from areas with comparable climatic and genetic characteristics, such as portions of the Middle East and North Africa (MENA). For example, a study conducted in Egypt found that the prevalence of PCOS was 13.5% among those who were fertile and 37.5% among those who were infertile [22], which is quite similar to rates in GCC countries. These similarities could be explained by common eating patterns, urbanization patterns, and high consanguinity rates [23]. One significant risk factor in the GCC is consanguinity, which may increase the genetic susceptibility to endocrine abnormalities that show up as hirsutism [24].

Underreporting remains a significant challenge in the GCC, driven by cultural stigmas and healthcare system barriers [25,26]. Conditions that impact physical appearance, especially those deemed cosmetic, are frequently taboo in conservative communities [27]. Because of peer pressure or a fear of being judged, many women may be ashamed to report symptoms or seek medical help [28]. Furthermore, especially in primary care settings, healthcare professionals might not be trained to recognize or prioritize hirsutism as a clinical problem [29]. The creation of successful public health initiatives is hampered by the gaps in epidemiological data caused by this underreporting.

Regional epidemiological research employing standardized approaches is needed to fill these gaps. A more precise assessment of the prevalence of hirsutism and its underlying causes can be obtained using population-based surveys that incorporate imaging tests, hormone profiling, and clinical examinations [30]. By involving local residents in the study process, community-based participatory research techniques can aid in overcoming cultural barriers [31,32]. Furthermore, longitudinal studies that monitor the occurrence and development of hirsutism and associated endocrine diseases over time would provide important information on their risk factors and natural history [33]. To carry out these programs, cooperation between legislators, researchers, and healthcare professionals is crucial. By providing funds for extensive research and incorporating hirsutism into national health agendas, governments can play a crucial role [34]. Destigmatizing the illness and educating women about the value of early diagnosis and treatment should be the goals of public health initiatives [30]. Improving diagnosis and treatment rates also requires addressing inequalities in healthcare access, especially for low-income and expatriate communities [35]. Furthermore, establishing collaborations with neighborhood organizations can improve outreach initiatives and guarantee that educational materials are accessed by the people most impacted by hirsutism and related conditions [36].

The GCC can strategically develop and implement well-targeted interventions that are specifically designed to tackle these issues head-on in an effective manner by effectively addressing and bridging the significant knowledge gaps that currently exist within the healthcare realm, particularly with regard to hirsutism and its associated underlying disorders. In addition to improving the personal health outcomes of those impacted by these conditions, this proactive approach will be crucial in helping achieve larger public health goals, such as lowering the burden of metabolic complications of obesity that are common throughout the region.

### 3.2. Cultural and Psychosocial Dimensions

Hirsutism can have serious psychosocial repercussions in the GCC’s culturally conservative societies, where physical appearance has a big impact on social acceptance and marriage prospects [37]. Low self-esteem, social isolation, and mental health issues, including anxiety and depression, are common among hirsute women [38]. Social beauty standards that value smooth, hairless skin exacerbate these problems and encourage women to seek cosmetic procedures as a first resort rather than treating the underlying medical causes [39].

Research has shown that the stigma associated with hirsutism is increased in the GCC due to cultural norms. For example, studies in Saudi Arabia and the United Arab Emirates have revealed that hirsute women often experience emotions of inadequacy and dread of being judged in social and familial contexts [8,40]. Qualitative interviews conducted in Oman also showed that women frequently experience direct or indirect pressure from family members to have cosmetic procedures done, which adds to their stress levels [41]. This ongoing pressure affects mental health and perpetuates the cycle of dissatisfaction with natural appearances, leading many people to select short-term cosmetic fixes rather than concentrating on long-term acceptance and self-love. In addition to perpetuating the stigma, the significant influence of beauty standards in these countries puts women under pressure to blend in, often at the expense of their mental health and sense of value in the process [42].

Traditional treatments like turmeric masks, which are popular and socially acceptable but lack scientific backing, are frequently used as coping mechanisms [43]. Despite their cultural resonance, these approaches have the potential to worsen the disease and postpone medical assistance [44]. According to the results of a survey conducted in Kuwait, the majority of respondents preferred these traditional ways over medical treatments since they were regarded to be safer and less expensive [45].

The role of media and public figures in shaping beauty standards cannot be overstated [46,47]. The psychological load on women with hirsutism is increased by social media platforms, which are frequently dominated by influencers who promote hairless looks [48]. These narratives can be changed by campaigns that highlight relatable role models who value their natural appearance [49]. In a recent Saudi Arabian initiative, healthcare organizations and local celebrities worked together to raise awareness of hormonal health, which resulted in more clinic visits and early diagnosis [50]. In addition to empowering women to ask for assistance, this cooperative approach dispels the stigma attached to hirsutism and promotes a more inclusive conversation about health and beauty. Women with PCOS-induced hirsutism have a substantial psychological burden, according to a Saudi Arabian study that emphasized the importance of support networks in reducing stress, anxiety, and depression [16]. The findings suggest that fostering safe spaces where women can talk about their struggles and experiences can be extremely important for improving mental health and encouraging acceptance of different ideals of beauty.

Reducing stigma and encouraging early identification and treatment requires increasing knowledge of hirsutism as a medical disorder rather than just a cosmetic problem. By promoting awareness and providing correct information, public health initiatives are essential in reducing the stigma associated with diseases like PCOS, which is a prevalent cause of hirsutism [51]. Furthermore, it is critical to combat stigma in healthcare institutions since it can compromise beneficial health outcomes, diagnosis, and treatment [52]. Putting mechanisms in place to lessen stigma is essential to providing high-quality healthcare. Involving community and religious leaders in these initiatives can also increase their legitimacy and create a positive atmosphere [53]. Peer-led support groups, for example, have been demonstrated to improve coping abilities, lower depressive symptoms, and improve health-related quality of life in women with PCOS [54]. Furthermore, since hirsutism can have serious psychological repercussions and lower quality of life, it is imperative to treat its psychosocial influence [55].

These initiatives not only empower individuals to seek help but also promote a broader understanding of the condition within society, ultimately reducing isolation and fostering acceptance.

### 3.3. Pathomechanism of Hirsutism and Molecular Basis

**Figure 2:**
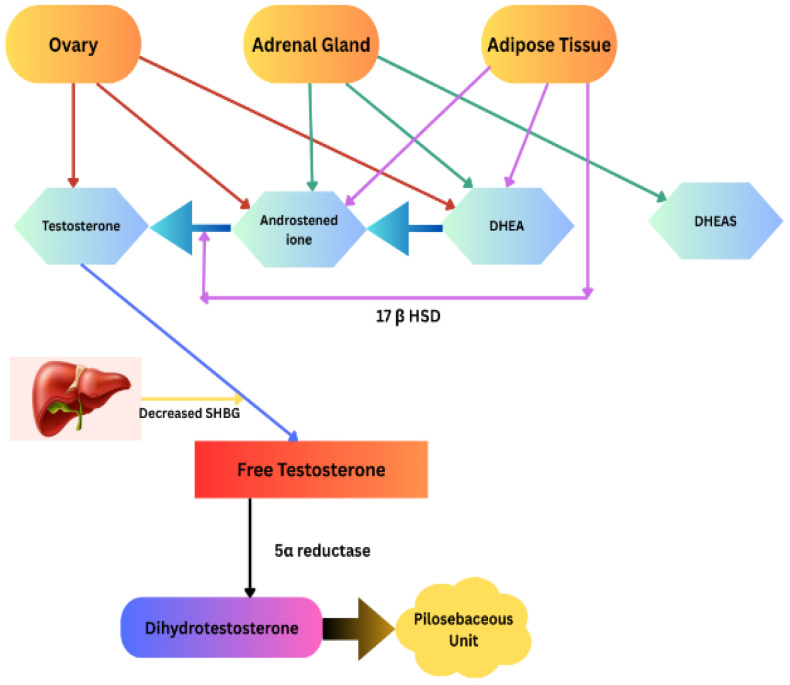
Sources of androgen in the female body and pathomechanism of hirsutism. [SHBG—serum hormone-binding globulin; 17β-HSD—17β-hydroxysteroid dehydrogenase].

The pathomechanism of hirsutism mainly involves androgen excess and sensitivity of hair follicles to androgens. Androgens control the activity of hair follicles by binding to androgen receptors in the dermal papilla, causing vellus hair to convert to terminal hair. Hirsutism can be a result of androgen excess and/or the individual’s response to androgens. Excess androgens enlarge and prolong the growth phase of hair follicles and make them grow dark, thick hairs on the face, chest, and back [56,57]. There seems to be variation among individuals of the pilosebaceous units in response to androgen levels. The common androgens in females are testosterone, dehydro-epi-androstenedione (DHEA), and dehydro-epi-androstenedione sulfate (DHEAS). Organs like the ovary, adrenal glands, and peripheral adipose tissue produce specific androgens and contribute to total androgens in the body (Figure 2). Of the total testosterone in females, only about one percent is free, and the rest is bound to serum hormone-binding globulin (SHBG). Obesity, insulin, serum testosterone, and exogenous androgens can reduce SHBG, increasing serum free testosterone levels [58]. Free testosterone is converted to dihydrotestosterone (DHT) by the enzyme 5 alpha reductase, and this DHT is responsible for terminal hair growth [59]. Various tissues in the body have different thresholds of androgens for terminal hair growth. Hair follicles have higher sensitivity to androgen in specific areas like the axilla and pubic region in females, and the terminal hair growth normally occurs at puberty. However, other areas have a higher threshold for this, and hirsutism usually occurs at higher levels of free testosterone. DHEA, DHEA-S, and androstenedione are considered pre-androgens and have less potent action on androgen receptors. However, as the levels of testosterone are low in women, they can play a role in the development of hirsutism [60]. DHEA-S can be used as a precursor by ovarian follicles to produce DHEA, testosterone, and DHT [61]. DHEA-S levels are increased by prolactin and insulin-like growth factor 1, which may explain the hyperandrogenism associated with other disorders [61].

There are different conditions clinically presenting with hirsutism in females. The specific conditions can have varied clinical and demographic profiles. Hence, the approach to hirsutism diagnosis and management should be contextual, considering all these factors. The disorders that usually present with hirsutism are listed below:

- **Adrenal causes**
  - Congenital adrenal hyperplasia (classic and non-classic)
  - Glucocorticoid resistance, cortisone reductase deficiency
  - Adrenal adenoma, carcinoma, bilateral macronodular adrenal hyperplasia
- **Ovarian causes**
  - PCOS
  - Ovarian tumors: Sertoli–Leydig cell tumors, granulosa-theca cell tumors, hilus cell tumors, hyperthecosis, rarely functioning teratoma, Krukenberg tumors
- **Idiopathic Hirsutism and Functional Hyperandrogenism**
- **Exogenous exposure**
- **Other endocrine diseases**
  - Hyperprolactinemia
  - Cushing’s disease
  - Acromegaly
  - Obesity and other insulin resistance syndromes
- **Medications** (danazol, valproic acid, oxcarbazepine)
- **Gestational** (luteoma of pregnancy, hyperreactio luteinalis)

#### 3.3.1. Polycystic Ovary Syndrome (PCOS)

The most common cause of hyperandrogenism, PCOS, encompasses ovarian dysfunction, resistance to insulin, and hypersecretion of the luteinizing hormone (LH), leading to hyperandrogenism. Hirsutism is additionally caused by the commonly associated irregularity of menstrual periods, anovulation, and metabolic disturbance that increases in women with PCOS. Advances in the molecular etiology of reproductive disorders show the pivotal role of epigenetic dysregulations, including aberrant imprinted gene methylation, a phenomenon that has been extensively reported on in the cases of gestational trophoblastic diseases [62]. Such mechanisms involving disrupted genomic imprinting, the anomalous methylation of dominant regulating genes, and the dysregulation of androgen-related pathways might underpin the aetiopathogenesis of hirsutism and PCOS even among genetically susceptible individuals.

About 19 loci have been identified near various genes that are associated with PCOS. They are located in chromosomes 2, 5, 8, 9, 11, 12, 16, 20, and 23. The five steroidogenic enzymes are cytochrome P450 side chain cleavage enzyme encoded by *CYP11A1* (cytochrome P450, family 11, subfamily A, member 1), 21 α hydroxylase (encoded by the *CYP21A2* gene: cytochrome P450 family 21 subfamily A member 2), 11 β hydroxylase (encoded by the *CYP11B1* gene: cytochrome P450, family 11, subfamily B, member 1), 3β-hydroxysteroid dehydrogenase 2 (encoded by the *HSD3B2* gene), and 17-hydroxylase/17, 20-lyase deficiency (encoded by the *CYP17A1* gene: cytochrome P450, family 17, subfamily A, member 1 [63] (Table 1).

#### 3.3.2. ACTH-Dependent Cushing’s Syndrome (Cushing’s Disease)

Cushing syndrome is either exogenous—usually the result of chronic glucocorticoid administration—or endogenous. Endogenous types are either ACTH-dependent (e.g., Cushing’s disease associated with pituitary adenomas) or ACTH-independent (e.g., adrenal adenomas or hyperplasia). The latter is of particular relevance to hirsutism through direct adrenal androgen excess. Prolonged exposure to elevated cortisol levels, either secondary to chronic glucocorticoid therapy or adrenal neoplasms, usually leads to HPA axis dysregulation and the subsequent hyperproduction of androgens. This condition presents with central obesity, insulin resistance, and clinical manifestations of hyperandrogenism, including hirsutism. Cushing’s disease is a state of hyperandrogenism due to pituitary tumors producing an excess amount of adrenocorticotropic hormone.

#### 3.3.3. Androgen-Secreting Tumors

Rare but of clinical significance, adrenal or ovarian tumors can cause sudden hyperandrogenism, widespread hirsutism, deepening of the voice, and other features of virilization. These tumors require immediate medical evaluation due to their virulence.

#### 3.3.4. Idiopathic Hyperandrogenism

In some cases, hyperandrogenism is not explained by identifiable ovarian or adrenal dysfunction. This condition, idiopathic hyperandrogenism (IH), suggests a genetic or environmental component in modifying androgen metabolism. The etiology of IH is largely unknown. Several mechanisms are put forth to explain IH. It is suggested that there may be hypersecretion of ovarian and adrenal androgens in IH [functional hyperandrogenism (FH)]. Functional hyperandrogenism is composed of various organ system-specific entities like ovarian or adrenal FH. Organ-specific FHs may be unrelated or complexly intertwined to manifest as different disorders. Functional adrenal hyperandrogenism (FAH) is generally defined as ACTH-dependent 17-ketosteroid excess, which is suppressible by glucocorticoids. It is proposed that FAH is due to increased adrenal sensitivity to ACTH at the level of the adrenal gland [70]. Functional ovarian hyperandrogenism (FOH), on the other hand, is a dysregulation of steroidogenesis as a result of intrinsic theca cell dysfunction or adrenocortical androgenic dysfunction [71]. Studies have also pointed out that variances in androgen receptors leading to hyperactivity even in the presence of normal serum androgen levels, higher activity of 5α-reductase locally increasing the levels of DHT, and decreased aromatase activities at the skin leading to relative local hyperandrogenemia can be alternative explanations [69]. Among the other theories, increased local androgen production by the pilosebaceous unit and an unexplained role of insulin resistance have been recognized [69].

#### 3.3.5. Obesity-Related Insulin Resistance and Obesity Metabolic Complications

Insulin resistance, a hallmark of metabolic diseases like type 1 and type 2 diabetes mellitus, features prominently in the worsening of hyperandrogenism and the clinical expression of hirsutism. The downregulation of sex hormone-binding globulin by hyperinsulinemia enhances free circulating androgens and leads to excess terminal hair growth. This increases the bioavailability of free testosterone, resulting in hirsutism. Lifestyle factors, including sedentary behavior and unhealthy eating patterns, which are becoming more common in GCC nations, have an impact on these hormone imbalances [72,73]. Growing rates of metabolic syndrome and obesity in the area greatly increase insulin resistance, which feeds back into hyperandrogenism [74]. Recent work has also highlighted the fact that even with insulin deficiency as a feature of type 1 diabetes mellitus, secondary metabolic dysregulation confers susceptibility to the same endocrine derangements [75]. Although type 1 diabetes is primarily autoimmune and features absolute insulin deficiency, compensatory hyperinsulinemia from insulin therapy and associated metabolic disruptions can lower SHBG and increase free androgens, thus exacerbating hirsutism.

According to Ruiz-Ojeda et al. [76], inflammation and oxidative stress are also important factors in the aggravation of hirsutism and hyperandrogenism. According to studies, androgen synthesis can be increased by persistent low-grade inflammation, which is frequently linked to obesity and metabolic diseases [77]. For example, studies in Oman have shown that women with PCOS have greater levels of oxidative stress indicators, which are associated with more severe hirsutism [78]. According to these results, tailored anti-inflammatory medications may be used in addition to conventional hormone therapies.

#### 3.3.6. Other Insulin-Resistant Syndromes (IRSs)

These are a heterogeneous group of rare disorders characterized by profound IR, metabolic abnormalities, and an array of clinical manifestations and complications. While obesity is the most common acquired IRS, other entities are mostly congenital. They include insulin receptor defects, signaling defects, and lipodystrophies. The rare disorders associated with clinical manifestations of hirsutism are Alström syndrome and type A and B insulin resistance syndromes. Rarer still are Donohue syndrome and Rabson–Mendenhall syndrome, which present with polycystic ovary morphology and mild hirsutism. Therefore, it is suggested that the detection of IR should be a part evaluation of hirsutism. Further genetic testing may be required in the presence of marked IR, especially in younger females with a family history of any of the disorders. When detected, the treatment includes lifestyle management, insulin sensitizers, lipid-lowering agents, and immunosuppressants in a few cases [79].

#### 3.3.7. Hyperprolactinemia

Hyperprolactinemia secondary to pituitary adenomas or drug intake can induce mild hyperandrogenism due to the inhibition of gonadotropin-releasing hormone (GnRH) secretion, with indirect increases in the production of adrenal androgens.

### 3.4. Pathomechanism of Hirsutism in GCC

Variations in the prevalence and severity of hirsutism may be caused by specific genetic and environmental factors in the GCC. The region’s widespread practice of consanguinity has been connected to a higher prevalence of genetic disorders that impact testosterone metabolism [80]. Hormonal imbalances may also be made worse by environmental exposures, such as eating a diet high in refined carbs and lacking in micronutrients [81]. Dietary therapies aimed at lowering glycemic load considerably improved androgen profiles in women with PCOS, according to a systematic review, highlighting the possibility of culturally specific lifestyle changes [82]. Although certain dietary habits do not directly contribute to hyperandrogenism, they indirectly lead to the development of obesity. Subsequently, obesity-related insulin resistance decreases SHBG levels, enhances free androgen bioavailability, and eventually causes hyperandrogenism and hirsutism.

Studies on hyperandrogenism conducted worldwide offer insightful information; however, they must be contextualized for the GCC population. For example, although Western research highlights the need for exercise in treating PCOS-related hirsutism [83], the GCC’s cultural and climatic conditions call for different strategies, like community-based therapies and indoor physical activities. The potential advantages of combining traditional dietary practices with contemporary medical treatments are further supported by evidence from South Asian cultures, which have some genetic and dietary similarities to populations in the GCC [84], emphasizing the value of a comprehensive strategy that addresses health issues while honoring cultural customs.

Even with these developments, there are still a lot of unanswered questions about the endocrine foundations of hirsutism in GCC nations. Longitudinal studies that fully explore and analyze the intricate relationships and interactions between genetic predispositions, diverse environmental influences, and the myriad of lifestyle decisions people make throughout their lives must be a major focus of future research initiatives. Furthermore, incorporating cutting-edge diagnostic techniques like hormone profiling and genetic screening into standard clinical practice may improve early detection and individualized treatment plans.

### 3.5. Screening and Diagnosis

There are various obstacles to hirsutism screening and diagnosis in GCC nations. Women in rural areas are neglected since access to specialized endocrinologists and advanced diagnostic technologies is frequently limited to urban centers [13,23]. Reproductive health-related cultural stigmas deter women from obtaining medical care, which delays diagnosis and results in the underreporting of cases [85].

The Ferriman–Gallwey (FG) scoring system is a crucial diagnostic tool that evaluates the degree of terminal hair development in nine androgen-sensitive regions [86]. However, the application of this tool in many ethnic groups is limited. According to studies, the FG rating system’s accuracy for GCC populations may be increased by making adjustments based on ethnicity [87]. To mitigate this issue and improve the dependability of FG scoring, diagnostic thresholds that are specific to a given region should be created and scores should be interpreted in the context of ethnicity. While abnormal scores are ≥2 in Han Chinese women, it is ≥9 in Middle Eastern, Mediterranean, South Asian, and Hispanic women [88].

Early detection rates could be considerably increased by incorporating hirsutism assessments into regular medical examinations. Gynecologists and primary care doctors should receive training on how to identify the symptoms of hirsutism and related endocrine problems [89]. In primary care settings, basic screening procedures like patient questionnaires and eye examinations could be used to find those who are at risk [90]. Research on the prevalence and knowledge of PCOS among Syrian women, for example, shows that raising awareness and regular screening programs are essential for enhancing comprehension and the early diagnosis of conditions (like PCOS) frequently linked to hirsutism [91]. These results highlight the importance of applying comparable techniques in GCC nations to improve early diagnosis and the treatment of PCOS-related hirsutism since Syrian women and GCC women share genetic commonalities [92].

Initiatives for community-based health also have the potential to raise awareness and encourage screening. In rural locations, mobile health units with skilled staff could offer screening services, closing the access gap to healthcare [93]. Additionally, women can be empowered to seek prompt medical attention by using digital channels to provide educational content regarding hirsutism and its causes [94]. For example, a pilot study in Tabuk, Saudi Arabia, assessed how well a private social network (PCOS system) educated women with PCOS about managing their condition [95].

Adopting standardized diagnostic criteria and educating medical professionals to provide culturally competent care should be the main goals of future initiatives. For women with hirsutism, complete therapy could be provided by establishing multidisciplinary clinics that integrate dermatology, endocrinology, gynecology, and mental health services. To guarantee fair access to care, policymakers must also address systemic obstacles, including the high expense of diagnostic testing and inadequate insurance coverage. Healthcare systems in the GCC can improve early intervention and outcomes for women with hirsutism by taking a proactive approach to screening and diagnosis.

### 3.6. Management and Treatment Approaches

Treating obesity is a cornerstone in the reduction of hirsutism, including the conditions associated with PCOS and the metabolic syndrome. Treatments involving calorie-restricted diets, physical training, and behavior modification achieve a reduction in body weight, decreased insulin resistance, and, in turn, lower levels of free androgens in the bloodstream. Pharmacological measures like metformin and GLP-1 receptor agonists are also effective in enhancing insulin sensitivity and endocrine balance. Bariatric surgery has shown a significant reduction in hyperandrogenic symptoms in the selected instances. Combining these strategies with the treatment of hirsutism will improve the outcomes considerably.

#### 3.6.1. Pharmacological Treatments

Hormonal medications are frequently used to treat hirsutism, which frequently results from underlying endocrine abnormalities [96]. According to studies, oral contraceptives are commonly recommended to control menstrual cycles and lower testosterone levels, which helps to prevent excessive hair growth [97,98]. Another essential treatment is anti-androgens, including spironolactone, which reduce hair follicle activation and inhibit androgen receptors [99]. Insulin-sensitizing medications, such as metformin, are used to improve metabolic profiles and indirectly lessen the severity of hirsutism in conditions where insulin resistance secondary to obesity is a central factor in the pathogenesis of PCOS [100].

#### 3.6.2. Non-Pharmacological Interventions

In the GCC, cosmetic operations have become increasingly popular as a way to treat hirsutism [101]. The authors conducted a cross-sectional survey involving Middle Eastern youth (n = 1200), assessing cosmetic treatment perceptions. Because of its low invasiveness and long-lasting effects, laser hair removal, which uses focused light radiation to kill hair follicles, is one of the most popular treatments [102]. By using electric currents to destroy individual hair follicles, electrolysis offers a permanent cure, despite taking more time [103]. This study reported outcomes from 35,000 h of electro-epilation (electrolysis) treatment on facial and neck areas in women with hirsutism, providing robust clinical insights into long-term efficacy and patient satisfaction with this method. According to a regional study carried out in Saudi Arabia, females with hirsutism favored laser hair removal over alternative cosmetic procedures since it produced better outcomes and had fewer adverse effects [104]. This study analyzed data from Saudi cosmetic surgery patients over a multi-year period and found that a growing number of younger female patients are seeking aesthetic treatments, reflecting changing social norms and increasing acceptance of cosmetic procedures. These techniques meet the high aesthetic standards that are common in the area, but some patients may find them prohibitive due to their expense and need for numerous sessions. Lifestyle interventions, including dietary changes, increased physical activity, and behavioral therapy, are considered important strategies in managing PCOS and associated hirsutism. These approaches can improve insulin sensitivity, reduce free androgen levels, and lead to clinically meaningful improvements in hirsutism.

#### 3.6.3. Role of Alternative Medicine

In the GCC, alternative medicine and traditional therapies are still culturally significant [105]. The possible anti-androgenic effects of herbal remedies, such as spearmint tea, have been investigated [106,107]. Furthermore, patients may seek therapies based on Ayurvedic and Unani systems, indicating a preference for natural and culturally recognizable methods [108,109]. However, these treatments’ safety and effectiveness are frequently not well supported by up-to-date scientific evidence, which calls for more research and patient education.

#### 3.6.4. Patient Compliance and Challenges

A key component of effectively managing hirsutism is adherence to treatment plans [110]. Consistent use may be hampered by issues including the cost of pharmaceutical treatments, especially for expatriates without full health insurance [111]. Patients may also decide not to continue therapy if they experience side effects such as skin irritation, nausea, or weight gain [112]. Furthermore, people may be deterred from obtaining medical or cosmetic procedures in conservative communities due to the stigma attached to hirsutism [113]. According to a systematic study that looked at mental health literacy in GCC nations, stigma around mental health problems is pervasive and can even spread to other medical diseases like hirsutism. The survey revealed that participants, including medical professionals, had low levels of mental health literacy, underscoring the need for more awareness and education to combat stigma and enhance healthcare experiences for individuals with conditions like hirsutism [114].

### 3.7. Public Health and Policy Implications

#### 3.7.1. National Health Strategies

National strategies for health in GCC countries are now more focused on fighting non-communicable diseases (NCDs) such as obesity and its complication, type 2 diabetes, which often coexist with hirsutism [115]. These may be expanded to incorporate the management of hirsutism based on its very roots, that is, hormonal imbalance and a spectrum of conditions such as metabolic syndrome, insulin resistance, type 2 diabetes mellitus, and dyslipidemia, all of which are interlinked with hormonal imbalances contributing to hirsutism. For example, a healthy, lifestyle-based approach to living, which also reduces obesity, can have positive, indirect effects on hirsutism by the improvement of insulin sensitivity [116,117]. To achieve this, healthcare professionals will need to partner with policymakers in order to help design focused education for patients and practitioners about hirsutism, its origins, and ways of treating the condition.

#### 3.7.2. Policy Examples from GCC Countries

Saudi Arabia’s Vision 2030 health reform [118] and the UAE’s National Strategy for Wellbeing 2031 [119] place a heavy emphasis on the role of preventive care and women’s health. These frameworks may offer a model whereby the management of hirsutism could be incorporated into more general NCD prevention programs. Policies aimed at facilitating access to endocrinological and dermatological care, for example, could be expanded to subsidized treatments for hirsutism, particularly for low-income groups.

#### 3.7.3. Public Health Campaigns

Successful public health campaigns for women’s health in the GCC include awareness drives for breast cancer and maternal health programs [120,121]. The strategies pursued by these campaigns, such as community outreach and the use of social media, can be leveraged in creating awareness about hirsutism, reducing associated stigma, and seeking timely medical consultations.

#### 3.7.4. International Collaborations

Collaboration with international entities like the WHO or the International Society of Endocrinology might offer additional resources and complement the local expertise in the region [122]. Joint funding can be arranged for research studies, capacity building, and even the development of uniform clinical guidelines, which would also be suitable both culturally and for GCC health contexts.

### 3.8. Future Directions

#### 3.8.1. Artificial Intelligence and Machine Learning

Newer technologies like artificial intelligence (AI) and machine learning (ML) hold great promise in diagnosis and management in hirsutism [123]. AI-powered diagnostic tools would identify endocrine disorders underlying the disease with much greater precision, while ML algorithms might make treatment protocols more personalized in patient-specific data, thus improving outcomes.

#### 3.8.2. Patient-Reported Outcome Measures (PROMs)

The use of patient-reported outcome measures (PROMs) in everyday clinical practice may provide valid data on the actual effectiveness of treatments as perceived by the patients themselves [124]. PROMs may help clinicians with individualizing intervention strategies to best meet patients’ needs and enhance overall patient satisfaction.

#### 3.8.3. Regional Research Networks

A regional research network within the GCC could standardize research protocols, share data, or promote collaborative studies regarding hirsutism and its related conditions. This would also help in building a strong evidence base for the development of effective interventions.

#### 3.8.4. Educational Materials and Training Programs

Cultural education about hirsutism and training for healthcare workers are recommended and will lead to improved diagnosis and treatment. The recommendations could also be guidelines regarding culturally sensitive communications with patients about hirsutism, current options for the most effective therapies, and practices of addressing stigma within clinical management. 

## Figures and Tables

**Table 1:** Genes implicated in the pathophysiology of hirsutism.

Disorder	Location in Chromosomes	Identified Genes [Reference]	Possible Mechanism
PCOS	2, 5, 8, 9, 11, 12, 16, 20, and 23	Loci near *PLGRKT*, *ZBTB16*, *MAPRE1, THADA, ERBB4, IRF1/RAD50*, *GATA4/NEIL2*, *FANCC*, *TOX3*, *DENND1A*, *YAP1*, *ARSD*, *ARL14EP/FSHB*, *SOD2*, *KRR1*, *ERBB3/RAB5B*, and *C9orf3* [64].	Identified variants were associated with hyperandrogenism, gonadotropin regulation, and testosterone levels. *THADA*, *FSHβ*, and *IRF1/RAD50* loci are associated with testosterone levels or regulation. *DENND1A* is associated with hyperandrogenism. *SOD2*, *ERBB3/RAB5*, *TOX3*, and *C9orf3* are associated with hyperandrogenism.
CAH	1, 6, and 8	*CYP21A2*, *CYP11B1*, and *HSD3B2*—associated with 21-hydroxylase, 11-beta-hydroxylase, and 3-beta-hydroxysteroid dehydrogenase enzyme deficiencies. *CYP21A2* and *CYP21A1* (pseudogene) mutations [63,65].	Due to a large gene deletion and conversion. A total of 32 variants of *CYP21A2*, 9 variants of *CYP11B1*, and 6 variants of *HSD3B2*. The mutations comprise promoter region mutations, intronic mutations, frameshift mutations, and single base pair missense mutations.
ACTH-dependent Cushing’s syndrome (Cushing’s Disease)	15, pseudo- genes in 2 and 8	Somatic mutations in the ubiquitin-specific protease 8 (*USP8*) gene increase the activity of the enzyme [66].	Dysregulation of ACTH synthesis and secretion caused by corticotroph tumors. Excessive deubiquitination of epidermal growth factor receptor (EGFR) tyrosine kinase disturbs its degradation [1]. EGFR expression, the overexpression of cyclin E (cell-cycle regulator), and low expression levels of the tumor protein p27 (cell-cycle inhibitor) are seen.
Cushing syndrome (endo genous ACTH-independent or exogenous)	1, 2, 5, 11, 16, 17, and 20	Bilateral hyperplasia due to *PRKAR1A* germline-inactivating mutations and macronodular hyperplasia by germline-inactivating mutations of *MEN1*, *APC*, *FH*, and *ARMC5* [67]. Others include *GNAS*, *PRKACB*, *PDE11A*, and *PDE8B* [68].	These mutations affect the cAMP/PKA/MAPK and Wnt signaling systems for the presentations. Increased production of ACTH leads to hyperandrogenism due to excessive production by the adrenal gland.
Adrenal adenoma	19	*PRKACA* somatic-activating mutations [67].	
Idiopathic hirsutism		Local androgen synthesis by the pilosebaceous unit [69].	Higher expression of steroid sulfatase and 17-beta hydroxysteroid dehydrogenase mRNA in skin.

A glossary of the genes can be found at the end of the text.

## Glossary of Genes

*PLGRKT*	Plasminogen Receptor with a C-Terminal Lysine
*ZBTB16*	Zinc Finger and BTB Domain Containing 16
*MAPRE1*	Microtubule-Associated Protein RP/EB Family Member 1
*THADA*	Thyroid Adenoma-Associated Protein
*ERBB4*	Receptor Protein–Tyrosine Kinase ErbB-4
*IRF1/RAD50*	Interferon Regulatory Factor 1/DNA Repair Protein
*GATA4/NEIL2*	GATA Binding Protein 4/Nei-like DNA Glycosylase 2
*FANCC*	Fanconi Anemia Complementation Group C
*TOX3*	TOX High-Mobility Group Box Family Member 3
*DENND1A*	DENN Domain Containing 1A
*YAP1*	Yes-Associated Protein 1
*ARSD*	Arylsulfatase D
*ARL14EP/FSHB*	ARF-like GTPase 14 Effector Protein/Follicle-Stimulating Hormone Subunit Beta
*SOD2*	Superoxide Dismutase 2
*KRR1*	KRR1 Small Subunit Processome Component Homolog
*ERBB3/RAB5B*	Erb-B2 Receptor Tyrosine Kinase 3/Ras-Related Protein Rab-5B
*C9orf3*	Chromosome 9 Open Reading Frame 3/Aminopeptidase O
*CYP21A1/CYP21A2*	Cytochrome P450 Family 21 Subfamily A Member 1/2
*CYP11 B1*	Cytochrome P450 Family 11 Subfamily B Member 1
*HSD3B2*	Hydroxy-delta-5-steroid dehydrogenase, 3 beta-, and Steroid Delta-isomerase 2
*USP8*	Ubiquitin-Specific Protease 8
*PRKAR1A*	Protein Kinase CAMP-Dependent Type I Regulatory Subunit Alpha
*MEN1*	Multiple Endocrine Neoplasia, Type 1
*APC*	Adenomatous Polyposis Coli
*FH*	Fumarate Hydratase
*ARMC5*	Armadillo Repeat Containing 5
*GNAS complex*	Guanine Nucleotide-Binding Protein, Alpha-Stimulating Activity Polypeptide
*PRKACA*	Protein Kinase CAMP-Activated Catalytic Subunit Alpha
*PRKACB*	Protein Kinase cAMP-Activated Catalytic Subunit Beta
*PDE11A*	Phosphodiesterase 11A
*PDE8B*	Phosphodiesterase 8B
